# Mobile phone use and risk of acoustic neuroma: results of the Interphone case–control study in five North European countries

**DOI:** 10.1038/sj.bjc.6602764

**Published:** 2005-08-30

**Authors:** M J Schoemaker, A J Swerdlow, A Ahlbom, A Auvinen, K G Blaasaas, E Cardis, H Collatz Christensen, M Feychting, S J Hepworth, C Johansen, L Klæboe, S Lönn, P A McKinney, K Muir, J Raitanen, T Salminen, J Thomsen, T Tynes

**Affiliations:** 1 Section of Epidemiology, Institute of Cancer Research, Brookes Lawley Building, Sutton SM2 5NG, UK; 2 Institute of Environmental Medicine, Karolinska Institute, Box 210, 171 77, Stockholm, Sweden; 3 STUK-Radiation and Nuclear Safety Authority, 00881 Helsinki, Finland; 4 Norwegian Armed Forces, Bygning 0028A, Sessvollmoen 2058, Norway; 5 International Agency for Research on Cancer, 150 Cours Albert Thomas, 69372 Cedex 08, Lyon, France; 6 Institute of Cancer Epidemiology, Danish Cancer Society, Strandboulevarden 49, 2100 Copenhagen, Denmark; 7 Centre for Epidemiology and Biostatistics, University of Leeds, 30 Hyde Terrace, Leeds LS2 9LN, UK; 8 The Cancer Registry of Norway, Institute of Population-based Cancer Research, Montebello, 0310 Oslo, Norway; 9 Division of Epidemiology and Public Health, University of Nottingham, Nottingham NG7 2UH, UK; 10 Tampere School of Public Health, University of Tampere, Tampere 33014, Finland; 11 Department of Otolaryngology-Head and Neck Surgery, Gentofte Hospital, University of Copenhagen, DK-2900 Hellerup, Denmark; 12 Norwegian Radiation Protection Authority, PO Box 55, 1332 Osteras, Norway

**Keywords:** neuroma, acoustic, telephone, epidemiology, aetiology

## Abstract

There is public concern that use of mobile phones could increase the risk of brain tumours. If such an effect exists, acoustic neuroma would be of particular concern because of the proximity of the acoustic nerve to the handset. We conducted, to a shared protocol, six population-based case–control studies in four Nordic countries and the UK to assess the risk of acoustic neuroma in relation to mobile phone use. Data were collected by personal interview from 678 cases of acoustic neuroma and 3553 controls. The risk of acoustic neuroma in relation to regular mobile phone use in the pooled data set was not raised (odds ratio (OR)=0.9, 95% confidence interval (CI): 0.7–1.1). There was no association of risk with duration of use, lifetime cumulative hours of use or number of calls, for phone use overall or for analogue or digital phones separately. Risk of a tumour on the same side of the head as reported phone use was raised for use for 10 years or longer (OR=1.8, 95% CI: 1.1–3.1). The study suggests that there is no substantial risk of acoustic neuroma in the first decade after starting mobile phone use. However, an increase in risk after longer term use or after a longer lag period could not be ruled out.

More than 1 billion people use mobile phones worldwide, with numbers growing rapidly (NRPB, 2004). There is public concern about a possible increased risk of brain tumours in mobile phone users, despite the absence of a known biological mechanism by which radiofrequency (RF) fields from the phones might cause neoplasms (IEGMP, 2000; AGNIR, 2003). In contrast to ionising radiation, RF fields do not have enough energy to break chemical bonds or damage DNA (AGNIR, 2003).

Acoustic neuroma (vestibular schwannoma) is a nerve sheath tumour of the vestibulocochlear nerve. This tumour is of particular interest in relation to mobile phones because brain deposition of energy from RF fields from a mobile phone is mainly within a small area of the skull close to the handset, which includes the vestibular portion of the eighth cranial nerve where acoustic neuromas develop (Rothman *et al*, 1996; Dimbylow and Mann, 1999). Some previous epidemiological studies on mobile phone use have suggested an increased risk of acoustic neuroma (Hardell *et al*, 2003; Lönn *et al*, 2004), but others have not (Hardell *et al*, 1999; Inskip *et al*, 2001; Johansen *et al*, 2001; Muscat *et al*, 2002; Warren *et al*, 2003; Christensen *et al*, 2004). The studies to date have been small (13–159 cases), however, with few long-term users and wide confidence intervals (CIs).

We undertook six population-based case–control studies within the Interphone collaboration (Cardis and Kilkenny, 1999) to investigate the risk of acoustic neuroma in relation to mobile phone use, with 678 cases and 3553 controls, in the UK and four Nordic countries. These countries were the first in Western Europe to introduce mobile phones and to have widespread use of these devices (Editorial, 1996). They are therefore especially suitable for the investigation of risks of tumours in relation to long-term mobile phone use.

## MATERIALS AND METHODS

### Study design and recruitment

Six population-based case–control studies of acoustic neuroma aetiology were conducted in the Nordic countries and the UK. The Nordic studies were conducted in Denmark nationwide, Finland excluding Northern Lapland and Åland, the Southern and middle regions of Norway, and the Stockholm, Göteborg and Lund regions of Sweden. One UK study was conducted in the Thames regions of Southeast England, and the other in Southern Scotland, the West Midlands, West Yorkshire and the Trent area. The Danish and Swedish studies have been reported previously (Christensen *et al*, 2004; Lönn *et al*, 2004). All studies followed the core protocol of the Interphone Study, coordinated by the International Agency for Research on Cancer (Cardis and Kilkenny, 1999) but with several extensions including a wider age range and hence extra subjects.

Cases were identified through neurosurgery, neuropathology, oncology, neurology and otorhinolaryngology centres in the study areas. Lists of cases were also obtained from the appropriate population-based cancer registries to ensure completeness of ascertainment. Eligible cases were individuals diagnosed with acoustic neuroma between 1 September 1999 and 31 August 2004 (the exact dates within this period vary by centre) at ages 20–69 years in the Nordic countries, 18–59 in Southeast England, and 18–69 in the Northern UK, and resident in the study region at the time of diagnosis.

Controls in the Nordic centres were randomly selected from the population register for each study area, frequency matched to cases on age, sex and region. In the UK, where there is no such accessible population register, controls were randomly selected from general practitioners’ practice lists. Controls were subject to the same age and residence criteria as cases and had never been diagnosed with a brain tumour.

Subjects were invited by letter to participate in the study. If no reply was received a repeat letter was sent or the subject was contacted by telephone. Each study was approved by the appropriate local ethics committee. Informed consent was obtained from all subjects at interview.

### Data collection

Trained interviewers administered a personal interview, which was usually conducted at the subject's home, the hospital or another place convenient for the subject. For almost half the interviews in Norway, however, and a small minority elsewhere, where face-to-face interviews were not possible, interviews were conducted over the telephone. The interview was computer-assisted, with the answers being entered directly into the questionnaire program on a laptop computer, except in Finland, where answers were recorded on a paper copy of the questionnaire and later entered into the program.

During the interview, participants were asked about use of mobile phones, and about models and makes of phones they had used. Subjects were shown photographs of the phones to aid their recall of this information.

For each phone model, information was collected on the start and end date of use, the average amount of time of use and number of calls. If any substantial changes in use were reported that lasted for more than 6 months, usage information was also collected for these periods. Data were collected on mobile phone network operator, the extent of hands-free use, whether the phone had mainly been used in rural areas, urban areas or both, the side of the head on which the phone was mainly used, and whether the subject was left or right handed.

### Statistical analysis

These studies were conducted as part of larger case–control studies on several types of intracranial tumour, with controls recruited for the entire set of cases. To increase statistical power, we used as controls for this analysis all participants interviewed as controls for the study who fitted the frequency matching strata of the acoustic neuroma cases.

In the calculation of all exposure indices, except time since first use, any phone use less than 12 months prior to diagnosis was excluded because it was most unlikely to be aetiologically relevant. Time since first use was evaluated up to the diagnosis date. Regular phone use was defined as having used a mobile phone for at least 6 months more than 1 year prior to diagnosis. As controls were not individually matched to cases, we constructed a date equivalent to the cases’ diagnosis date to truncate exposure for controls. We refer to the date of diagnosis or equivalent date for controls as the ‘reference date’. For controls this was obtained by constructing, for each centre, case-strata by single calendar year of interview and single year interval between diagnosis and interview (‘interview lag time’). Controls interviewed in each calendar year were randomly allocated to strata of interview lag time, proportionally to the distribution of the cases in the same calendar year, to obtain a similar distribution of lag time as in the cases. The reference dates for controls were then calculated by subtracting the mean interview lag time in cases in that stratum from the interview dates of the controls.

We calculated lifetime cumulative numbers of hours of phone use and numbers of calls by summing the calculated numbers of hours of use and numbers of calls for each usage pattern of each phone model. Risks for cumulative use were analysed with and without modification for reported use of headsets and/or hands-free sets in a vehicle, using methods described elsewhere (Christensen *et al*, 2004; Lönn *et al*, 2004).

Analyses were performed by individual centre and on the overall pooled data set. Odds ratios (ORs) were calculated as estimates of relative risk, and were obtained using conditional logistic regression, with strata of centre, region, 5-year age group at reference date and sex, and adjusted for highest educational level and combinations of interview year and interview lag time. Heterogeneity in results between centres was assessed with a log likelihood ratio test comparing a model with an interaction between centre and the exposure with a model with only the main effects. We also conducted parallel analyses using a two-stage random-effects model (Stukel *et al*, 2001); these are not presented because they gave very similar results and there was no between-centre heterogeneity (defining heterogeneity, conservatively, as *P*<0.10). The statistical package STATA was used for these analyses (StataCorp, 2003). All statistical tests were two sided.

We conducted analyses of laterality of the tumour in relation to the reported side of the head that the phone was predominantly held, using the methods of Inskip (Inskip *et al*, 2001) and of Lönn *et al* (2004). The first method assesses the association between side of phone use and laterality of the tumour in cases only. In the second method, reported use ipsilateral (or contralateral) to the tumour is analysed in right- and left-sided tumour cases separately, with 50% of controls randomly assigned to each group, and the two ORs, with adjustment for confounding factors, are pooled. As these analyses are dependent on random allocation of controls, we repeated the random allocation 300 times by simulation to check for the consistency of reported results, which were found to be reasonably robust. Subjects who reported using the phone equally often on each side of the head were treated as ‘exposed’ in both the ipsilateral and contralateral analyses; we also repeated the analysis excluding this group as a background check on whether it affected the results. Owing to the potential for the reported side of phone use to be influenced by recall bias, we also analysed the relation of tumour laterality to side of handedness, using the same methods as for reported side of phone use.

As exposure to ionising radiation is a known risk factor for acoustic neuroma, we repeated the analysis after excluding subjects who reported having had radiotherapy more than 10 years prior to the reference date.

## RESULTS

A total of 827 potential cases and 8460 potential controls were mailed an invitation letter for the study. The participation rate was 83% (range between centres 69–91%) among cases and 51% (42–69%) among controls mailed, and 84% (71–93%) and 61% (55–76%), respectively, based on individuals definitely receiving the letters. The main reasons for nonparticipation were refusal (cases 9%, controls 28%), inability to contact subjects (cases 2%, controls 18%), no permission from the doctor to approach the subject (cases 3%, controls 2%) and illness or death (cases 1%, controls 0%). In total, 684 cases were interviewed, of whom six were excluded because they had neurofibromatosis, leaving 678 cases in the analysis. The number of controls interviewed was 4340, of whom 3553 corresponded to matching strata of the cases and were included in the analysis. The numbers from each centre are shown in Table 1. In all, 95% of cases and 96% of controls were interviewed face-to-face, and the remainder over the telephone.

The relative risk of acoustic neuroma for regular mobile phone use was 0.9 (95% CI: 0.7–1.1) (Table 2). Centre-specific relative risks for regular phone use were 0.6 (95% CI: 0.3–1.4) for Norway, 0.6 (95% CI: 0.4–1.1) for Finland, 0.8 (95% CI: 0.5–1.3) for Denmark, 0.9 (95% CI: 0.6–1.4) for Sweden, 1.0 (95% CI: 0.7–1.6) for Southeast England and 1.1 (95% CI: 0.7–1.6) for the Northern UK. There was no significant heterogeneity of these relative risks between centres (*P*=0.50), or for any of the other results, so centre-specific results are not presented, for brevity. Using a 5-year instead of a 1-year latency period, the relative risk in the pooled data set was 1.0 (95% CI: 0.8–1.3) (data not shown).

Risk of acoustic neuroma did not increase with increasing time since first regular phone use or lifetime number of years of use (Table 2). For first use more than 10 years ago, the relative risk was 1.0 (95% CI: 0.7–1.5) the greatest centre-specific risk, as previously reported (Lönn *et al*, 2004), was in Sweden (OR=1.6, 95 % CI 0.8–3.4), and nonsignificant relative risks ranged from 0.4 to 1.3 for the other centres (data not shown).

There was no trend in risk with lifetime cumulative hours of phone use or cumulative number of calls (Table 2). The relative risk was 1.0 (95% CI: 0.7–1.3) for the top quartile of cumulative number of calls and 0.9 (95% CI: 0.7–1.2) for the top quartile of cumulative hours of use compared with never or nonregular users. Cumulative hours of use 10 years or more prior to the reference date showed a relative risk of 1.2 (95% CI: 0.8–2.0) for 90 or more cumulative hours of use (90=median) compared with never or nonregular use. Reanalysis of the cumulative exposure estimates adjusting for use of hands-free devices did not materially alter the results (data not shown).

Regular use of analogue, or of digital (GSM), phones showed no association with risk. There was no association with time since first use, number of years of use or lifetime hours of use for either type of phone (Table 3). The relative risk for more than 82 h (82=median) of analogue use in the period 10 or more years prior to the reference date was 1.5 (95% CI: 0.9–2.5) (data not shown).

The relative risk of a tumour ipsilateral to the side of reported phone use in regular mobile phone users was 0.9 (95% CI: 0.7–1.1) using the method described by Lönn *et al* (2004) (Table 4). First use 10 or more years ago showed a relative risk for ipsilateral tumours of 1.3 (95% CI: 0.8–2.0) and the ipsilateral relative risk for 10 or more cumulative years of use was 1.8 (95% CI: 1.1–3.1). Ipsilateral relative risks were not appreciably raised, or were reduced, for more recent and for shorter periods of use. Relative risks of contralateral tumours were generally slightly above unity. Repeating the laterality analyses after excluding subjects reporting bilateral use gave a near identical result for 10 or more years of ipsilateral use (OR=1.8, 95% CI: 1.0–3.3), but a lower relative risk for 10 or more years of contralateral use (OR=0.7, 95% CI: 0.3–1.6) (data not shown). Using the analysis method of Inskip *et al* (2001), the overall relative risk of a tumour ipsilateral to use of the phone was 0.9 (Fisher's exact test: *P*=0.4), the risk 10 or more years after start of ipsilateral use was 1.5 (*P*=0.08), and the risk for 10 or more years of cumulative ipsilateral use was 1.8 (*P*=0.09).

The relative risk of a tumour ipsilateral to handedness was 1.0 (95% CI: 0.8–1.3) in regular mobile phone users, and there was some suggestion of a trend in risk with increasing cumulative years of use by ipsilateral handed subjects (*P* trend=0.09). The relative risk for 10 or more years of use in these subjects was 1.5 (95% CI: 0.8–2.7) (data not shown).

In total, 89% of cases and 24% of controls responded positively to the question: ‘Do you suffer from any loss of hearing?’ Cases reported experiencing hearing loss on average 5.5 years before diagnosis and controls 13.3 years before the reference date. Among controls with no hearing loss, 59% of regular phone users reported predominantly right-sided use, 33% left-sided use and 8% use on both sides. Among cases, there was a less pronounced preference of side (49, 40 and 11%, respectively). Reported side of use among cases was more often contralateral than ipsilateral to the tumour in short-term phone users and the proportion of cases reporting use on both sides was highest in long-term users.

Based on the distribution of preferred side of phone use among controls, if mobile phones cause acoustic neuromas, one might expect a higher proportion of tumours on the right than on the left side of the head among regular phone users. The proportion of right-sided tumours was 53.3% in regular users compared with 49.3% in never or nonregular users (*P* Fisher's exact test=0.34). In regular analogue phone users, the proportion of right-sided tumours was 58.8% (*P*=0.13) and in regular digital phone users, it was 52.4% (*P*=0.5). However, there was a nonsignificant deficit of right-sided tumours in long-term phone users overall (48.8%) and in long-term analogue phone users (48.7%) (data not shown).

Eight cases and 16 controls had had radiotherapy to the skull 10 or more years before the reference date. Excluding these subjects from the analysis did not materially change the results.

## DISCUSSION

There is public anxiety about possible cancer risks from mobile phone use. Although no such effect has been established to date, if it existed it would be of significance because of the high prevalence of use – there are currently more than 1 billion mobile phone users worldwide (NRPB, 2004). If mobile phones can cause acoustic neuroma, one would expect an increased risk of acoustic neuroma in mobile phone users after a certain latency period, and an increasing risk in relation to cumulative duration and frequency of use. Depending on the mechanism, one might expect the risk to be highest for analogue phone use, because these phones have a higher average power output than digital phones (IEGMP, 2000) and because they have been in use the longest. Furthermore, because the energy from the RF fields is absorbed superficially close to the handset (Dimbylow and Mann, 1999), the risk should be highest on the side of the head on which the phone is used.

In our analyses based on several times as many cases and controls as previously published, from five North European countries where mobile phones were introduced early, risk of acoustic neuroma was not raised for regular mobile phone use. Furthermore, there were no significantly raised risks in relation to number of years of use, time since first use, cumulative hours of use or cumulative number of calls, or separately for analogue or digital phones. There was a significantly raised risk for reported mobile phone use for 10 or more years ipsilateral to the tumour, but risks were not raised for shorter durations of ipsilateral use nor for overall ipsilateral use. Self-reported side of phone use, however, is potentially an extremely biased variable (Boice and McLaughlin, 2002; Health Council of the Netherlands, 2002; Rothman, 2001), especially when asked about after development of the tumour.

First, hearing loss produced by the tumour could cause the user to change use to the other ear, even before the tumour is definitely diagnosed, leading to an underestimation of ipsilateral risk. Consistent with this, we found that in short-term users, phone use by cases was more likely to be contralateral than ipsilateral, and in long-term users (who would have started phone use before the tumour first began to develop), reported bilateral phone use was more common than in short-term users. Secondly, recall bias could distort the findings in the opposite direction: cases might over-report ipsilateral use because they believe it caused their tumour. This bias would be expected not just to raise risks for ipsilateral use but also to reduce apparent risks for contralateral use (Rothman, 2001). Contralateral risks were not in general appreciably reduced in our data, although there was some evidence of a reduced risk in the analyses of long-term use after excluding bilateral users. A third potential bias is that the tumour might be detected earlier in people who use the phone on the same side as the developing tumour, because they notice the hearing loss sooner than people who use the phone on the other side. This would result in increased risks for short-term as well as long-term phone users, but no increased risk for short-term users was found in our study. Overall, given the multiple, potentially contrary sources of bias no firm conclusions can be drawn from the analyses on side of use.

We analysed tumour laterality by handedness as an alternative marker of actual side of use. Handedness has the advantage that it is unlikely to be subject to recall bias, but has the disadvantage that not all people use the phone on their handed side. Risk of acoustic neuroma ipsilateral to side of handedness was nonsignificantly raised after 10 or more cumulative years of phone use – compatible with, but not giving strong support to, the results on reported side of use.

No trend in risk was found in relation to cumulative hours of phone use or number of calls. These measures, however, are subject to substantial misclassification in recall (Parslow *et al*, 2003), which would dilute any real effect.

Among the eight previously reported studies on risks of acoustic neuroma and mobile phone use (Hardell *et al*, 1999, 2003; Inskip *et al*, 2001; Johansen *et al*, 2001; Muscat *et al*, 2002; Warren *et al*, 2003; Lönn *et al*, 2004; Christensen *et al*, 2004), two showed any significant raised risks. A study by Hardell *et al* (2003) showed a raised risk with use of analogue phones (OR=4.4, 95% CI: 2.1–9.2), which was raised for both ipsilateral and contralateral use. This is the only study that reported an increased risk in short-term users, but it has been heavily criticised for methodological limitations (Rothman, 2000, 2001; Boice and McLaughlin, 2002; AGNIR, 2003; Ahlbom *et al*, 2004). The second study (Lönn *et al*, 2004), which is part of the present analysis, reported a somewhat raised risk (OR=1.9, 95% CI: 0.9–4.1) 10 years after the start of mobile phone use, which was significantly increased (OR=3.9, 95% CI: 1.6–9.5) when restricted to tumours ipsilateral to reported phone use. The results in the present analysis for the Swedish centre are slightly different from those in the previous publication because of differences in analytical approach, but are in the same direction. The six published studies that did not show evidence of raised risk had few long-term users.

There is potential bias because acoustic neuroma is a very slow growing tumour (Thomsen and Tos, 1990) and symptoms of the tumour, in particular hearing loss, experienced long before diagnosis could have influenced subjects’ behaviours regarding phone use. This is a plausible reason for the somewhat reduced relative risk in relation to regular phone use overall, with a relative risk of 1.0 after excluding phone use during the 5 years prior to diagnosis.

There is also potential for selection bias among controls. The overall participation rate among cases was in the range of 83–84% and among controls was 51–61%, depending on the proportion of nonrespondents who actually received an invitation. Much effort was invested to maximise control participation rates by several rounds of follow-up after the initial invitation letter. To prevent selection bias, the study was introduced to potential study participants at some centres as a study about general lifestyle risk factors for cancer, and at some as a study of the causes of brain tumours, depending on the local ethics agreements. In the Swedish and Finnish studies, enquiries to obtain restricted data (over the telephone) from nonparticipants found a somewhat lower prevalence of phone use in those willing to give such data than in full participants (Lönn *et al*, 2004; Lahkola *et al*, 2005), but these results are of uncertain significance because only a minority of nonparticipants replied. Generally, however, the lack of raised risk of acoustic neuroma in relation to phone use overall was seen in our analyses for centres with higher as well as those with lower control participation rates.

Confounding by known risks factors is unlikely to explain our results. Neurofibromatosis type II is associated with the tumour, but is rare, and we excluded cases with this condition from the study. High-dose ionising radiation is the only established environmental risk factor (Ron *et al*, 1988; Preston *et al*, 2002), but results were not altered by excluding patients who received radiotherapy to the head.

In summary, our findings do not support an increased risk of acoustic neuroma in the first decade after starting mobile phone use. For 10 or more years of use, there was an increased risk of tumours ipsilateral to reported phone use, of uncertain interpretation. There is no consistent biological evidence that exposure to RF fields is implicated in the development of tumours nor has a potential aetiological mechanism been demonstrated (IEGMP, 2000; AGNIR, 2003). Overall, there is no convincing epidemiological evidence that RF exposure is related to neoplasia (Ahlbom *et al*, 2004). Thus on balance, the evidence suggests that there is no substantial risk of acoustic neuroma in the first decade of use, but the possibility of some effect after longer periods remains open.

## Figures and Tables

**Table 1:** Characteristics of acoustic neuroma cases and controls

	**Cases (*n*=678)**		**Controls (*n*=3553)**	
**Characteristic**	***N***	**%**	***N***	**%**
*Sex*				
Male	318	46.9	1646	46.3
Female	360	53.1	1907	53.7
				
*Age at reference date (years)*				
18–29	36	5.3	178	5.0
30–39	121	17.9	511	14.4
40–49	171	25.2	880	24.8
50–59	255	37.6	1358	38.2
60–69	95	14.0	626	17.6
				
*Centre*				
Denmark	102	15.0	782	22.0
Finland	91	13.4	572	16.1
Norway	45	6.6	254	7.2
Sweden	144	21.2	479	13.5
UK-North	133	19.6	892	25.1
UK-Southeast England	163	24.0	574	16.2
				
*Highest school level completed*				
Primary school	124	18.3	765	21.5
Secondary/high school	195	28.8	992	27.9
Medium level technical/professional	167	24.6	908	25.6
University/higher level technical	190	28.0	882	24.8
Not known	2	0.3	6	0.2
				

**Table 2:** Risks of acoustic neuroma in relation to various characteristics of mobile phone use

	**Cases**		**Controls**		
**Factor**	**No.**	**%**	**No.**	**%**	**OR (95% CI)**
*Frequency of use*					
Never/nonregular use^a^	316	46.6	1612	45.4	1.0
Regular use	360	53.1	1934	54.4	0.9 (0.7–1.1)
Not known	2	0.3	7	0.2	
					
*Years since first use*					
Never/nonregular use^a^	316	46.6	1612	45.4	1.0
1.5–4^b^	174	25.7	1014	28.5	0.8 (0.7–1.0)
5–9	139	20.5	708	19.9	0.9 (0.7–1.2)
⩾10	47	6.9	212	6.0	1.0 (0.7–1.5)
Not known	2	0.3	7	0.2	*P* trend=0.9
					
*Lifetime years of use*					
Never/nonregular use^a^	316	46.6	1612	45.4	1.0
0.5–4	231	34.1	1270	35.7	0.9 (0.7–1.1)
5–9	96	14.2	515	14.5	0.9 (0.7–1.2)
⩾10	31	4.6	131	3.7	1.1 (0.7–1.8)
Not known	4	0.6	25	0.7	*P* trend=0.7
					
*Cumulative no. of calls* c					
Never/nonregular use^a^	316	46.6	1612	45.4	1.0
<2149	173	25.5	952	26.8	0.9 (0.7–1.1)
2149–8000	82	12.1	473	13.3	0.8 (0.6–1.1)
>8000	99	14.6	477	13.4	1.0 (0.7–1.3)
Not known	8	1.2	39	1.1	*P* trend=0.5
					
*Cumulative hrs. of use* c					
Never/nonregular use^a^	316	46.6	1612	45.4	1.0
<116	168	24.8	951	26.8	0.9 (0.7–1.1)
116–534	89	13.1	472	13.3	0.9 (0.7–1.2)
>534	94	13.9	476	13.4	0.9 (0.7–1.2)
Not known	11	1.6	42	1.2	*P* trend=0.5
					
*Cumulative hrs. of use by time since first use*					
Never/nonregular use^a^	316	46.6	1612	45.4	1.0
<10 years	313	46.2	1722	48.5	0.9 (0.7–1.1)
⩾10 years (<90 h)^d^	19	2.8	106	3.0	0.8 (0.5–1.4)
⩾10 years (⩾90 h)^d^	27	4.0	104	2.9	1.2 (0.8–2.0)
Not known	3	0.4	9	0.3	

a No use or nonregular use defined as user for total of less than 6 months in the period more than 1 year before the reference date.

b Lower limit 1.5 years ago because regular phone use defined as phone use of at least 6 months’ duration at least 1 year prior to the reference date.

c Classification based on median and quartiles among controls who were users: data divided into <median, median⩽third quartile and >third quartile.

d 90 h is the median number of hours of use 10 or more years prior to the reference date.

**Table 3:** Risks of acoustic neuroma in relation to various characteristics of analogue or digital phone use

	**Type of phone^a^**					
	**Analogue**			**Digital**		
**Factor**	**Cases**	**Controls**	**OR (95% CI)**	**Cases**	**Controls**	**OR (95% CI)**
*Frequency of use*						
Never/nonregular use^b^	316	1435	1.0	316	1609	1.0
Regular use	101	414	0.9 (0.7–1.2)	323	1770	0.9 (0.7–1.1)
						
*Years since first use*						
Never/nonregular use^b^	316	1435	1.0	316	1609	1.0
1.5–4^c^	10	58	0.5 (0.2–1.1)	208	1157	0.9 (0.7–1.1)
5–9	48	195	0.9 (0.6–1.3)	113	598	0.9 (0.7–1.2)
⩾10	43	161	1.1 (0.7–1.7)	2	15	0.7 (0.2–3.5)
			*P* trend=0.8			*P* trend=0.6
*Lifetime years of use*						
Never/nonregular use^b^	316	1435	1.0	316	1609	1.0
0.5–4	71	279	0.9 (0.7–1.3)	264	1413	0.9 (0.7–1.1)
5–9	22	107	0.8 (0.5–1.3)	58	348	0.9 (0.6–1.2)
⩾10	7	26	1.1 (0.4–2.8)		2	0 (0-)
Not known	1	2	*P* trend=0.7	1	7	*P* trend=0.5
						
*Cumulative hours of use* d						
Never/nonregular use^b^	316	1435	1.0	316	1609	1.0
<median	41	204	0.7 (0.5–1.1)	148	867	0.8 (0.7–1.1)
Median-3rd quartile	21	102	0.7 (0.4–1.2)	80	432	0.9 (0.7–1.2)
>3rd quartile	35	102	1.3 (0.8–2.0)	88	434	1.0 (0.7–1.3)
Not known	4	6	*P* trend=1.0	7	37	*P* trend=0.7

a Of the 5341 phones listed, for 223 (4.2%) the subject did not recall the model and 130 (2.4%) could not be coded to analogue or digital for other reasons. A total of 248 (10.8%) regular users had used one or more phones that could not be categorised. Repeating the analyses without these 248 subjects produced nearly identical results. For the analogue phone analysis, 260 cases and 1445 controls, and for the digital phone analysis 39 cases and 157 controls were excluded because they were only regular users of all phones combined or only of the digital or analogue type, respectively. A further one case and 259 controls for the analogue phone analysis and 17 controls for the digital phone analysis were excluded because they had no controls or cases, respectively, in their matching stratum.

b Reference group is never or nonregular use of mobile phones of any type.

c Lower limit 1.5 years ago because regular phone use defined as phone use of at least 6 months’ duration at least 1 year prior to the reference date.

d Classification based on the distribution among controls who were users. Data were divided into <135, 135–493, >493 for analogue phones, and <94, 94–388, >388 for digital phones.

**Table 4:** Risks of acoustic neuroma in relation to reported side of use of the mobile phone, by laterality of tumour

	**Side of tumour compared with reported side of phone use^a^**					
	**Ipsilateral**			**Contralateral**		
**Factor**	**Cases**	**Controls**	**OR (95% CI)**	**Cases**	**Controls**	**OR (95% CI)**
*Frequency of use*						
Reference category^b^	457	2444	1.0	446	2497	1.0
Regular use	187	1061	0.9 (0.7–1.1)	198	1008	1.1 (0.9–1.4)
						
*Years since first use*						
Reference category^b^	457	2444	1.0	446	2497	1.0
1.5–4^c^	82	557	0.7 (0.5–0.9)^d^	103	525	1.2 (0.9–1.5)
5–9	74	380	1.0 (0.7–1.3)	75	378	1.1 (0.8–1.5)
⩾10	31	124	1.3 (0.8–2.0)	20	105	1.0 (0.6–1.7)
			*P* trend=0.4			*P* trend=0.6
						
*Lifetime years of use*						
Reference category^b^	457	2444	1.0	446	2497	1.0
0.5–4	111	699	0.8 (0.6–1.0)^e^	133	654	1.2 (1.0–1.5)
5–9	51	281	0.9 (0.7–1.3)	53	270	1.1 (0.8–1.5)
⩾10	23	72	1.8 (1.1–3.1)^e^	12	73	0.9 (0.5–1.8)
Not known	2	9	*P* trend=0.11		11	*P* trend=0.9
						
*Cumulative hours of use* f						
Reference category^b^	457	2444	1.0	446	2497	1.0
<median	82	522	0.8 (0.6–1.0)	101	497	1.2 (0.9–1.6)
Median-3rd quartile	41	258	0.8 (0.6–1.2)	49	247	1.2 (0.8–1.6)
>3rd quartile	60	261	1.2 (0.8–1.6)	42	248	0.9 (0.6–1.3)
Not known	4	20	*P* trend=0.5	6	16	*P* trend=0.6

a A total of 29 cases excluded because tumour laterality was not known. Five cases and 11 controls excluded because of lack of phone use data. A total of 37 controls excluded because there were no cases in their matching stratum.

b The reference category is never or nonregular use of any type of mobile phone and, in the ipsilateral analysis, phone use only on the opposite side of the tumour, and in the contralateral analysis, phone use only on the same side as the tumour.

c Lower limit 1.5 years ago because regular phone use defined as phone use of at least 6 months’ duration at least 1 year prior to the reference date.

d *P*<0.01.

e *P*<0.05.

f Classification based on the distribution among controls who were users. Data were divided into <119, 119–593 and >593 for ipsilateral use, and <122, 122–534 and >534 for contralateral use.
